# Antifungal susceptibility profiles of otomycosis etiological agents in Ahvaz, Iran

**DOI:** 10.18502/CMM.6.2.2696

**Published:** 2020-06

**Authors:** Maral Gharaghani, Marzieh Halvaeezadeh, Gholam Ali Jalaee, Simin Taghipour, Neda Kiasat, Ali Zarei Mahmoudabadi

**Affiliations:** 1 Department of Medical Mycology, School of Medicine, Ahvaz Jundishapur University of Medical Sciences, Ahvaz, Iran; 2 Infectious and Tropical Diseases Research Center, Health Research Institute, Ahvaz Jundishapur University of Medical Sciences, Ahvaz, Iran; 3 Otorhinolaryngologist, Apadana Hospital, Sorosh Street, Ahvaz, Iran

**Keywords:** Antifungals, *Aspergillus* species, Caspofungin, Otomycosis

## Abstract

**Background and Purpose:**

Otomycosis is a secondary ear fungal infection among predisposed individuals in humid conditions. *Aspergillus* species are the most common etiologic agents of this infection. Several ototopical antifungals are currently used for the treatment of this disease; however, recurrence and treatment failure are usually observed in some cases. Regarding this, the present study was conducted to investigate the antifungal activity of caspofungin, azoles, and terbinafine against the isolated agents of otomycosis.

**Materials and Methods:**

This study was conducted on the specimens collected from 90 patients with otomycosis. The samples were cultured on Sabouraud dextrose agar and identified based on morphological characteristics, physiological tests, and microscopic features. Furthermore, the microdilution method was used for antifungal susceptibility testing according to the Clinical and Laboratory Standards Institute (CLSI) guidelines. Finally, the minimum inhibitory concentration (MIC) and minimum effective concentration (MEC) ranges, MIC/MEC_50_, MIC/MEC_90_, and geometric mean (GM) MIC/MEC were calculated for the isolates.

**Results:**

According to the results, 77 patients with otomycosis were positive for different *Aspergillus* (88.3%) and *Candida* (11.7%) species. *Aspergillus niger* complex (n=36) was found to be the most common agent, followed by *A. flavus, A. terreus,* and *A. nidulans* complexes. Furthermore, epidemiological cutoff values (ECVs) were lower than those presented by the CLSI for itraconazole and caspofungin in 98.5% and 42.6% of *Aspergillus* species, respectively. Terbinafine exhibited a great activity against *Aspergillus* species, while fluconazole revealed a low activity against both *Aspergillus* species. Based on the results, 77.8% of *Candida* species were resistant to caspofungin; however, miconazole and econazole had low MIC ranges.

**Conclusion:**

*Aspergillus niger* and *A. flavus* complexes were identified as the most common agents accounting for 85.7% of the isolates. In addition, terbinafine was identified as the best antifungal for both *Aspergillus* and *Candida* species. Moreover, tested azoles had relatively low MICs, whereas most of the isolates had the MIC values beyond the caspofungin ECVs.

## Introduction

Otomycosis, an acute to chronic fungal infection, is often considered a secondary outer ear fungal infection, including the auricle and external auditory canal. This disease also affects the tympanic membrane in some cases as a result of disease extension. However, the middle ear is rarely infected by the causative agents of otomycosis. The most common symptoms of this disease include itching, pain, redness, swelling, ear discharge, and ear fullness with gradual hearing loss. 

Although the anatomy of the ear canal acts as a route and residence of fungal elements, tropical conditions, trauma, bacterial otitis, ear anomalies, and hearing aids are predisposing factors for otomycosis [ 1 , 2 ]. On the other hand, low socioeconomic status and occupation (involving exposure to dusty and fungal contaminants) have an important role in the occurrence of this disease in a tropical climate [ 3 , 4 ]. Recently, diabetes, steroid administration, HIV infection, chemotherapy, several malignancies, and invasive therapies were also added to the list of otomycosis predisposing factors [ 5 ].

Several species of saprophytic fungi, including molds, yeasts, and rarely dermatophytes, are involved in the development of otomycosis. However, the species of the genus *Aspergillus*, especially *Aspergillus niger* complex, are the most common causative agents of this infection, with a worldwide distribution [ 3 , 6 ]. In a review study conducted on Iranian studies, Gharaghani et al. reported *A. niger* as the most common etiological agent of otomycosis (51.15%) in Iran, followed by *A. flavus* (17.03%), *Candida albicans* (11.39%), *A. fumigatus* (7.33%), and other species (13.1%) [ 3 , 7 - 9 ]. Although the majority of otomycosis cases are usually caused by *Aspergillus* species, different* Candida* species play an important role in the disease as endogenous organisms [ 7 , 8 ].* Candida* species have been reported as the second most common otomycosis agents; in this regard, *C. albicans* was recognized as the most common agent [ 4 , 8 , 10 ]. Furthermore, the rare cases of otomycosis due to dermatophytes and *Malassezia* species have been also reported by researchers [ 4 ]. 

The elimination and/or control of predisposing factors and antifungal therapy are necessary for the complete treatment of otomycosis. Topical antifungals, such as clotrimazole, miconazole, econazole, and nystatin, are specific agents for the treatment of otomycosis [ 11 , 12 ]. However, the increasing resistance to antifungals underscores the need for the development of new antifungals. Furthermore, the investigation of new antifungal drugs is an interesting area for many of the mycologists and clinicians. Caspofungin, a new antifungal with fungicidal activity, is used for the treatment of invasive aspergillosis [ 13 ] and candidiasis [ 14 ]. In addition, some studies have tested the activities of this antifungal against the different species of *Aspergillus* and obtained effective potential [ 15 - 17 ]. With this background in mind, the present study was conducted to isolate, identify, and study the antifungal activity of caspofungin, miconazole, fluconazole, terbinafine, econazole, bifonazole, and itraconazole against the causative agents of otomycosis.

## Materials and Methods

**Patients and sampling**

For the purpose of the study, 90 patients suspected of having otomycosis were physically examined by an otorhinolaryngologist. Clinical specimens (i.e., debris and pus) were collected from the patient’s ears using moisture swabs. The swabs were cultured on the slants of Sabouraud dextrose agar (SDA; Merck, Germany) and incubated at 27-29C for a week. Cultures were examined daily, and all grown fungi subcultured on two fresh SDA slants were preserved at ambient temperature until use. 

**Identification of causative agents**

All recovered fungi were primarily subcultured on fresh SDA slants. The yeast isolates were detected using germ tube formation, growth at 42-45C, morphology on Cornmeal agar (HighMedia, India) supplemented with 1% Tween 80 (Merck, Germany), and colony coloration on CHROMagar* Candida* (CHROMagar* Candida*, France). Moreover, the colony morphology characteristics and microscopic features were used for *Aspergillus* species. 

**Standard suspension preparation**

In order to prepare a standard suspension, each of the tested isolates was cultured on SDA for 48-72 h at 35°C. A suspension of spores was prepared in 0.85% NaCl, containing 0.05% Tween 20, adjusted to 0.5 McFarland according to the CLSI M38 3^rd^ edition, and then diluted at a ratio of 1:50 [ 18 ]. Moreover, a suspension of yeast colonies was prepared in sterile distilled water, adjusted to 0.5 McFarland based on the CLSI M27 4^th^ edition, and then diluted at a ratio of 1:1000 [ 19 ]. 

**Susceptibility assessment**

Antifungal susceptibility assay was performed based on the microdilution method (96-well microdilution plates), using the CLSI M38 3^rd^ edition and M27 4^th^ edition for molds and yeasts, respectively [ 18 , 19 ]. A serial dilution of antifungals was prepared in RPMI 1640 (Bio-Idea, Iran), including miconazole (0.0312-16 μg/mL), fluconazole (0.25-128 μg/mL), terbinafine (0.0156-8 μg/mL), caspofungin (0.0156-8 μg/mL), econazole (0.0156-8 μg/mL), bifonazole (0.0312-16 μg/mL), and itraconazole (0.0156-8 μg/mL). According to the CLSI instruction, 100 μL of each antifungal dilution and 100 μL of standard suspension were added to each well. Negative and positive controls were a well-contained RPMI 1640 without fungal spores and antifungals and a well-contained RPMI 1640 with fungal spores and without antifungals, respectively. 

The microplates were incubated at 35°C for 24 h and minimum inhibitory concentration (MIC) was read for 48 h (when the control well showed insufficient growth) for yeasts and molds. Then MIC range, MIC_50_, MIC_90_, and geometric mean (GM) MIC were calculated for the isolates. With regard to caspofungin, minimum effective concentration (MEC) range, MEC_50_, MEC_90_, and GM MEC were calculated for *Aspergillus* isolates. In the present study, susceptibility/resistance of the strains to each antifungal was evaluated according to the breakpoints and epidemiologic cutoff values (ECVs) defined by the CLSI and/or several researchers [ 20 - 22 ].

***Ethical consideration***

This study was approved by the Ethics Committee of Ahvaz Jundishapur University of Medical Sciences, Ahvaz, Iran (IR.AJUMS.REC.1394.118). In addition, all patients or parents signed a consent form before sampling. 

## Results

In the present study, 77 (85.6%) of the 90 specimens obtained from the patients with otomycosis were positive for different *Aspergillus* (n=68, 88.3%) and* Candida* (n=9, 11.7%) strains. A total of 36 isolates belonged to *A. niger* complex as the most common causative agent, followed by *A. flavus* complex (n=30), *A. terreus* complex (n=1), and *A. nidulans* complex (n=1). Moreover, 9 isolates of* Candida*, including *C. albicans*, *C. glabrata*, and* Candida* species (every 3 isolates), were identified in the samples.

Table 1 presents the in vitro antifungal susceptibility results of 7 antifungals against *Aspergillus* species. Terbinafine exhibited a great activity in vitro with the GM MIC values of 0.10509 and 0.17678 μg/mL against *A. niger* complex and *A. flavus* complex isolates, respectively. Moreover, MIC values for terbinafine ranged within 0.0.0312-1 μg/mL, indicating that all isolates were sensitive to this antifungal at ≥ 1 μg/mL. In the present study, 98.5% and 42.6% of *Aspergillus* species had ECVs lower than those presented by the CLSI for itraconazole and caspofungin, respectively [ 21 ]. In addition, fluconazole revealed a low activity against both *Aspergillus* species (i.e., *A. niger* and *A. flavus* complexes). In this respect, 27.8% and 46.7% of *A. niger* and *A. flavus* complexes were invariably resistant to fluconazole, respectively. Although there are no defined breakpoints or ECVs for miconazole, bifonazole, and econazole for *Aspergillus* species, the MIC range for these antifungals were relatively low.

**Table 1:** Antifungal susceptibility of otomycosis agents (*Aspergillus* species complex)

Species	Antifungals	Minimum Inhibitory Concentration (µg/mL)				Resistant (%)	%ECV^a^
		MIC	MIC_50_	MIC_90_	MICGM		
*Aspergillus niger* complex (36)	Terbinafine	0.0312-0.5	0.0625	0.5	0.10509	-	ND
	Caspofungin^b^	0.0312-8	0.0312	4	0.12444	15 (41.7%)	58.3%
	Fluconazole	1-128	64	128	50.79683	10 (27.8%)	ND
	Itraconazole	0.0312-2	0.5	1	0.39641	0 (0.0%)	100%
	Miconazole	0.25-4	0.25	2	0.39685	-	ND
	Bifonazole	0.0312-2	0.5	1	0.29671	-	ND
	Econazole	0.0625-0.5	0.0625	0.125	0.07577	-	ND
A. flavus complex (30)	Terbinafine	0.0625-1	0.25	0.5	0.17678	-	ND
	Caspofungin^b^	0.0312-8	8	8	3.24849	23 (76.7%)	23.3%
	Fluconazole	1-128	64	128	64	14 (46.7%)	ND
	Itraconazole	0.0312-8	0.25	1	0.30777	1 (3.3%)	96.7%
	Miconazole	0.25-8	1	4	1.02337	-	ND
	Bifonazole	0.0312-8	2	8	1.58732	-	ND
	Econazole	0.0312-0.25	0.125	0.25	0.10389	-	ND
A. terreus complex (1)	Terbinafine	0.25	-	-	-	-	ND
	Caspofungin^b^	8	-	-	-	1 (100%)	0.0%
	Fluconazole	16	-	-	-	0 (0.0%)	ND
	Itraconazole	0.25	-	-	-	0 (0.0%)	100%
	Miconazole	1	-	-	-	-	ND
	Bifonazole	0.5	-	-	-	-	ND
	Econazole	0.125	-	-	-	-	ND
A. nidulans complex (1)	Terbinafine	1	-	-	-	-	ND
	Caspofungin^b^	0.125	-	-	-	0 (0.0%)	100%
	Fluconazole	128	-	-	-	1 (100%)	ND
	Itraconazole	0.125	-	-	-	0 (0.0%)	100%
	Miconazole	1	-	-	-	-	ND
	Bifonazole	2	-	-	-	-	ND
	Econazole	0.125	-	-	-	-	ND

ECV: epidemiologic cutoff values, ND: not determined

^a^ %MICs less than or equal to the ECVs (ECV=1-2 µg/ml for itraconazole, 0.06-0.12 µg/ml for caspofungin)

^b^ Minimum effective concentration (MEC), MEC50, MEC90, and MECGM were calculated for organisms.

Table 2 presents the details related to the antifungal susceptibility of* Candida* species. According to the breakpoints presented by the CLSI [ 21 , 22 ], most of the isolates (77.8%) were resistant to caspofungin, followed by fluconazole (33.3%) and itraconazole (33.3%). Although both azoles (i.e., miconazole and econazole) had low MIC ranges (i.e., 0.25-4 and 0.0625-1 μg/mL, respectively), the MIC range for bifonazole was 2-16 µg/mL. 

**Table 2:** Antifungal susceptibility of otomycosis agents (*Candida* species)

Species	Antifungals	MIC	Resistant (%)
*Candida albicans* (3)	Terbinafine	4	0 (0.0%)
	Caspofungin	0.5-1	1 (33.3%)
	Fluconazole	4-16	2 (66.7%)
	Itraconazole	0.5-4	1 (33.3%)
	Miconazole	0.5-4	-
	Bifonazole	8-16	-
	Econazole	0.0625-0.5	-
*C. glabrata* (3)	Terbinafine	2-8	0 (0.0%)
	Caspofungin	0.5-2	3 (100%)
	Fluconazole	1-32	0 (0.0%)
	Itraconazole	0.0625-8	2 (66.7%)
	Miconazole	0.25-4	-
	Bifonazole	4-16	-
	Econazole	0.0625-1	-
*Candida species* (3)	Terbinafine	0.0625-1	0 (0.0%)
	Caspofungin	1-2	3 (100%)
	Fluconazole	2-16	1 (33.3%)
	Itraconazole	0.0625-0.25	0 (0.0%)
	Miconazole	2-4	-
	Bifonazole	2-8	-
	Econazole	0.0625-1	-
Total (9)	Terbinafine	0.0625-8	0 (0.0%)
	Caspofungin	0.5-2	7 (77.8%)
	Fluconazole	1-32	3 (33.3%)
	Itraconazole	0.0625-8	3 (33.3%)
	Miconazole	0.25-4	-
	Bifonazole	2-16	-
	Econazole	0.0625-1	-

## Discussion

Otomycosis is one of the problematic infectious diseases among tropical and subtropical inhabitants, posing diagnostic and therapeutic challenges to patients, clinicians, and laboratory workers. Several antifungal agents, including clotrimazole, miconazole, ketoconazole, itraconazole, lanoconazole, voriconazole, fluconazole, and nystatin, have been used for the treatment of otomycosis [ 9 , 10 , 23 , 24 ]. However, there are still unresponded/persistent and recurrent cases of otomycosis as reported by several researchers [ 24 , 25 ]. Moreover, the literature is indicative of the in vitro resistance of the causative agents of this disease against antifungal agents [ 26 , 27 ]. There are a few studies in the literature investigating the activity of terbinafine against *Aspergillus* species with otomycosis sources. In a study performed by Karaarslan et al., *Aspergillus* species (i.e., *A. niger*, A, flavus, and *A. terreus*) isolated from otomycosis was reported to exhibit a relatively low MIC range (0.03-.25 µg/mL) for terbinafine [ 28 ]. This observation agrees with our results showing the inhibition of *A. niger* complex strains by terbinafine at a MIC range of 0.0312-0.5 µg/mL. However, in the present study, the MIC range of *A. flavus* complex was slightly higher (0.0625-1 µg/mL). Alcazar-Fuoli et al. reported a low MIC value (0.07-1.17 µg/mL) for terbinafine against black *Aspergilli* obtained from different clinical specimens [ 29 ]. Moreover, in a study carried out by Zarei Mahmoudabadi et al., terbinafine was reported to be the best antifungal against the causative agent of otomycosis [ 30 ]. 

Different results have been reported for the efficacy of caspofungin against *Aspergillus* species. In the present study, 57.4% of *Aspergillus* isolates were resistant to caspofungin. Nonetheless, in a study performed by Pfaller et al., 100% of *Aspergillus* strains were inhibited by caspofungin at the MEC range of 0.007-0.12 μg/mL [ 31 ]. Moreover, Arikan et al. reported the MEC range of 0.25-1 μg/mL for caspofungin against different *Aspergillus* species [ 32 ]. In addition, our results revealed that 58.3% of *A. niger* complex had the ECVs lower than the values defined by CLSI in comparison to *A. flavus* complex (23.3%). 

Yenisehirli et al. and Szigeti et al. reported resistance to fluconazole in all of the *Aspergillus* strains recovered from otomycosis with a high MIC value (≥256 and >64 µg/mL) [ 6 , 33 ]. Nevertheless, in the current research, only 36.8% of our *Aspergillus* isolates were resistant to this azole. In addition, in our study, the MIC value ranges showed that *Aspergillus* species were inhibited by itraconazole (<8 µg/mL), and only one strain of *A. flavus* complex was resistant to this antifungal (≥8). Similarly, Yenisehirli et al. and Diekema et al. found that all of their *Aspergillus* species were sensitive to itraconazole [ 17 , 33 ]. Miconazole, bifonazole, and econazole had a low MIC range against *A. niger* complex, *A. terreus* complex, and *A. nidulans* complex strains. However, the MIC range was slightly higher for *A. flavus* complex isolates. Ahmed et al. showed that 76% of *Aspergillus* isolates from patients with otomycosis were sensitive to miconazole based on the disk diffusion method [ 34 ]. 

The present study involved the examination of a few strains of* Candida*, causing otomycosis, against several antifungals. The results revealed that most of the isolates (77.8%) were resistant to caspofungin. In a study carried out by Pfaller et al., 99.7% of* Candida* species (from blood or normally sterile sites) were inhibited at a caspofungin MIC of < 1 μg/mL [ 35 ]. It seems that the sources of isolation could affect their susceptibility to caspofungin. In line with the results obtained by Szigeti et al., our results showed that all* Candida* isolates were highly susceptible to terbinafine [ 6 ]. In addition, 33.3% of the tested isolates were resistant to both fluconazole and itraconazole, while the MIC ranges for econazole, miconazole, and bifonazole were low, medium, and high, respectively. 

## Conclusion

As the findings of the present study indicated, *A. niger* and *A. flavus* were identified as the most common otomycotic fungal pathogen complexes by accounting for 85.7% of the isolates. According to the susceptibility assay, terbinafine was the best antifungal for otomycosis agents (i.e., both *Aspergillus* and* Candida* species). Moreover, azoles, such as itraconazole, econazole, miconazole, and bifonazole, were found to have relatively low MICs. In contrast, most of the isolates were out of the defined ECVs for caspofungin. 
